# The association between smoking and family health with the mediation role of personality among Chinese people: nationwide cross-sectional study

**DOI:** 10.1186/s12888-024-05654-x

**Published:** 2024-03-14

**Authors:** Jiangyun Chen, Menglin Luo, Li Gan, Haomiao Li, Siyuan Liu, Na Ren, Yan Zhou, Jiao Yang, Haozheng Zhou, Xuanhao Yin, Jiahuan Wan, Xinlei Yang, Yibo Wu, Zenni Luo

**Affiliations:** 1 Department of Health Management, School of Health Management of Southern Medical University, Guangzhou, China; 2 Operation Management Department, Zhuhai People’s Hospital (Zhuhai Hospital Affiliated With Jinan University), Zhuhai, China; 3 School of Political Science and Public Administration, Wuhan University, Wuhan, China; 4 School of Public Health, Southern Medical University, Guangzhou, China; 5 School of Health Management, Capital Medical University, Beijing, China; 6 School of Public Health, Peking University, 38 Xueyuan Road, Haidian District, Beijing, 100191 China; 7 School of Health Management, Guangzhou Medical University, Xinzao, Panyu District, Guangzhou, China; 8 School of Pharmaceutical, Southern Medical University, Guangzhou, China

**Keywords:** Big Five personality, Smoke, Family health, Mediation, KHB mediated decomposition

## Abstract

**Background:**

There may be unexplored interactions between family health, personality, and smoking that could help provide new perspectives on tobacco control.

**Objective:**

To examine the relationship between the health of one’s family and their smoking habits, as well as investigate the potential influence of personality on this relationship.

**Methods:**

For this cross-sectional investigation, a national survey conducted in China in 2022 recruited a total of 21,916 individuals. The Family Health Scale was utilized to assess the health of the family. The 10-item Big Five Inventory scale was utilized to assess the Big five personality traits. The relationship between big five personality, family health, and smoking were investigated using binary and linear logistic regression. The indirect effects mediated by Big five personality were analyzed using mediation analysis with Sobel tests, and the indirect effects were composited using the Karlson-Holm-Breen method.

**Results:**

The overall prevalence of smoking in the study population was 14.87%, 26.19% for males and 3.54% for females. Urban and rural smoking prevalence was 13.81% and 16.10% respectively. Binary logistic regression analysis revealed a significant negative relationship between smoking and family health (odds ratio 0.964, 95% CI 0.959, 0.970, *P* < 0.001) with covariates controlled. The Karlson-Holm-Breen composition facilitated the connection between extraversion (47.81%) and nervousness (52.19%).

**Conclusions:**

Preventive interventions for smoking behavior should prioritize family health and the Big five personality as significant areas to focus on. According to this study, in addition to implementing various interventions for different personalities, family health should be strengthened to reduce smoking behavior.

**Supplementary Information:**

The online version contains supplementary material available at 10.1186/s12888-024-05654-x.

## Introduction

Smoking poses a significant risk of developing cardiovascular diseases, chronic respiratory diseases, diabetes, malignant tumors, and other life-threatening conditions [49, 50], which seriously undermine the health of the Chinese population. The tobacco epidemic has led to extensive health problems and an economic burden in China, necessitating urgent measures for smoking cessation. The “2030 Healthy China” Plan outlines the target of reducing the smoking rate among individuals over the age of 15 to less than 20% by 2030 [9]. Despite a series of tobacco control measures implemented by China [39], a significant gap still remains in achieving this goal. Smoking behavior is influenced by a combination of individual characteristics, psychological and behavioral factors [19], and situational factors. While traditional tobacco control strategies focus mainly on health education, tobacco tax, and tobacco control regulations, less attention is given to the “irrational factors” of smoking behavior and its underlying psychological mechanisms.

The smoking behavior of individuals is greatly influenced by the health of their family. The concept of ‘family health’ refers to a family-based health resource that develops from the intersection of individual family members’ health, daily interactions, self-efficacy, and the family’s social, emotional, economic, and medical resources [45]. This definition emphasizes the critical role of the family’s structure and interaction patterns in determining and shaping family members’ behavior. A supportive family environment can play a pivotal role in promoting health, such as by facilitating healthy lifestyle changes [27]. Family health strongly influences individual smoking behavior in terms of family structure, economic status, emotional support, and family member behavior [21, 37, 38].

Personality is a heritable and enduring psychological trait [26] that has garnered ongoing interest for its role in smoking behavior. One of the most widely accepted structural models of personality is the Big Five personality factor model [47]. Personality has been shown to be associated with both health-promoting practices and health risk behaviors [28]. Numerous studies have demonstrated that personality is linked with smoking behavior and is a significant predictor of smoking [11]. Specifically, daily smokers tend to exhibit higher neuroticism tendencies compared to former and never smokers [5]. Higher levels of neuroticism have been associated with emotional triggers for smoking [52] and poorer smoking cessation outcomes [52]. Additionally, lower levels of dutifulness have been found to predict smoking initiation in adulthood [5, 41, 52]. Other studies have reported that smokers have higher levels of extraversion and openness and lower levels of agreeableness than nonsmokers [5, 7].

Family health can have a significant impact on personality through various factors, including family structure, family relationships, family climate, and the socioeconomic status of the family [29, 36, 51]. Family-level factors can have a profound and long-lasting effect on the development and formation of an individual’s personality by influencing their level of mental health, resilience, emotional regulation, attitudes, knowledge, and behavioral empowerment. External social support from the family can also predict an individual’s extroverted personality and interpersonal interactions to some extent [20]. The impact of family health on personality is dynamic, and different types of family health environments that interact with the social environment can substantially influence personality formation and change [15].

Our previous study in China found that family health functioning and Big Five personality are both influential factors in adult smoking behavior [42], but the relationship between family health and personality was not considered. In summary, previous research has demonstrated that family health impacts individual smoking behavior, personality is a significant factor in smoking behavior, and family health may be related to the formation and change of personality. However, the relationship between these three factors remains unclear. Understanding the pathways between family health, personality, and smoking can provide a new perspective on tobacco control at the individual-family level in the context of “irrational factors” and is of great practical significance.

### The present study

This study collected data on family health, personality traits, and smoking behavior from a national survey with the aim of exploring the mediating role of Big five personality traits between family health and smoking behavior. Based on the above literature review, the following hypotheses were formulated:

- Hypothesis 1: Family health is associated with smoking behavior.
- Hypothesis 2: Personality traits are associated with family health and smoking behavior, respectively, and serve as a mediator in the association between family health and smoking behavior.

## Methods

### Data and procedure

Data for this research were gathered from a nationwide investigation carried out by the School of Public Health at Peking University between June 20th and August 31st, 2022. The survey covered 148 urban areas, 202 districts and counties, 390 townships, towns, or streets, and 780 communities or villages across 23 provinces, 5 autonomous regions, and 4 municipalities directly governed by the central government in China. The sampling rate was determined using the population proportion given in the data from the seventh national census. Individuals were chosen at various levels including municipal, district, county, township, town, street, and community, with quotas determined by gender and age. Researchers (*n* < 10) were enlisted from nearby colleges in every selected city and underwent training on sampling techniques, research tools, and quality assurance.

In order to be eligible for the research, individuals had to fulfill the following requirements: (1) be 12 years old or older; (2) hold citizenship in the People’s Republic of China; (3) have permanent residency in China (with no more than one month spent away from home annually); (4) willingly take part in the study and submit a signed informed consent document; (5) possess the ability to independently or with the assistance of the investigator complete the online survey; and (6) comprehend the significance conveyed in each item of the questionnaire. Individuals who had a mental disability or abnormality, cognitive dysfunction, were involved in other comparable research studies, or were uncooperative were not included as participants. A total of 23,414 questionnaires were collected, ensuring the data’s high quality and national representativeness. By eliminating duplicates, excluding missing data, and addressing outliers with logical inconsistencies, we obtained a final sample size of 21,916, which yielded a valid response rate of 93.6%. The survey protocol has been published [43].

### Measurements

#### Short-form of Family Health Scale (FHS-SF)

To comprehensively evaluate the extent of family health, the Family Health Scale (Short-Form) [8] was employed. Participants were inquired about their evaluation of the family health, such as ‘I experience a sense of security in my familial connections’. The scale consisted of 10 items that measured a single dimension, and each item was assessed using a Likert-5 scale that ranged from ‘strongly disagree’ (scored as 1) to ‘strongly agree’ (scored as 5). A higher score on the scale indicated a greater level of family health. The reliability and validity of this scale have been successfully validated. The scale has a Cronbach’s alpha coefficient of 0.849, while each dimension has a Cronbach’s alpha coefficient ranging from 0.762 to 0.915 (for detailed items, see Supplementary Table S3).

#### 10-item Big Five Inventory (BFI-10)

The scale includes five dimensions, namely extraversion, agreeableness, conscientiousness, nervousness, openness. Nervousness is defined by a tendency towards emotional volatility and self-awareness. A cognitive preference for creativity and aesthetics is what characterizes openness to experience. Interpersonal relationships are influenced by agreeableness and extraversion. Extraversion indicates a inclination for being sociable, enthusiastic, assertive, and seeking thrills, whereas agreeableness pertains to being warm, kind, gentle, trusting, and dependable. Conscientiousness is defined as the inclination to be responsible and skilled. Each dimension contains two items (10 items in total). All the questions in the scale are scored with Likert’s five-level scoring method. Each sub-scale has a maximum score of 10 points. As the score increases, the personality trait becomes increasingly evident. According to previous research, it has been demonstrated that it possesses favorable reliability and validity [34].

#### Smoking

The respondents’ smoking status is measured by the following questions: “Do you have the habit of smoking in the past month?” The answer options are: (1) Yes; (2) Have/have quit smoking; (3) No. Based on this question, smoking status is finally divided into two categories: (1) smokers: smokers now; (2) non-smokers: respondents who have never smoked or have quit smoking. See Supplementary Table 1 for variable descriptions and assignments.

#### Control variable

Confounders were chosen based on their correlation with the independent variable and their influence on the alteration of the relationship between the independent and dependent variables. To account for variations, age and gender were considered as constant factors to be accounted. The remaining variables were considered as potential confounders in the final models if they altered the estimates of family health on smoking by over 10% or had a significant association with the smoking core. The control variables chosen for examination included household type, place of residence, marital status, level of education, income, employment status, religious beliefs, political affiliation, presence of chronic diseases, experience of negative events, and level of depression, all of which were selected based on established associations and/or plausible biological relationships. Supplementary Tables 3, 4 and 5 provide a comprehensive breakdown of the connections between each confounding factor and smoking.

### Data analysis

The sociodemographic features and big five personality traits of the participants were grouped based on whether they smoked or not, and then summarized using frequencies (percentages) or means and standard deviations (SD). Our report presents the breakdown of smoking rates based on demographic attributes. To evaluate the correlation between family health, including its four aspects, big five personality rating, and smoking, binary logistic regressions were utilized while accounting for covariates and confounding variables. Odds Ratio (OR) with 95% CIs was calculated after adjusting for age, gender, household type, registered permanent residence, marital, education, income, work status, religion, politics, chronic diseases, negative event, and depression. The Sobel-Goodman Mediation Test was used to examine how the Big five personality traits (extraversion, agreeableness, conscientiousness, neuroticism, openness) mediate the relationship between family health (including social/emotional health processes, health resources, and external social supports) and smoking, while controlling for selected covariates. Karlson-Holm-Breen (KHB) techniques were employed to evaluate the impact magnitudes of mediators by combining the indirect effects with a logistic model.

Statistical significance was defined using an alpha level of 0.05 with 2-sided *p*-values. The analysis of the data was conducted using Stata (version 16) and Empower2.0.

## Results

### Characteristics of samples

The basic features of the 21,916 individuals are presented in Table 1, demonstrating an equal distribution of genders accounting for approximately half of the entire sample. Most of the populace resided in urban areas, accounting for 15,188 individuals or 69.3% of the total population. In the study population, the smoking rate was 14.87% in general, with males having a rate of 26.19% and females having a rate of 3.54%. Urban and rural smoking prevalence was 13.81% and 16.10% respectively. According to the data, smoking rates were found to be more common in males, older individuals, rural regions, individuals with one or multiple chronic illnesses, individuals with lower levels of education, individuals with lower income, and individuals experiencing depression (*P* < 0.001).

**Table 1:** Demographic distribution of smoking behavior

	**All**	**Currently smoking**		**Smoking prevalence rate (%)**	***P*-value**
		**Yes**	**No**		
**Number of participants**	21916	3258	18658	14.87	
**Age**					< 0.001
12–17 years	2072 (9.45)	118 (3.62)	1954 (10.47)	5.69	
18–59 years	15647 (71.40)	2405 (73.82)	13242 (70.97)	15.37	
≥ 60 years	4197 (19.15)	735 (22.56)	3462 (18.56)	17.51	
**Gender**					< 0.001
Male	10958 (50.00)	2870 (88.09)	8088 (43.35)	26.19	
Female	10958 (50.00)	388 (11.91)	10570 (56.65)	3.54	
**Religion**					0.045
None	21058 (96.09)	3110 (95.46)	17948 (96.19)	14.77	
Yes	858 (3.91)	148 (4.54)	710 (3.81)	17.25	
**Political landscape**					< 0.001
Party member or Probationary Party	3179 (14.51)	627 (19.24)	2552 (13.68)	19.72	
Member of the Communist Youth League	4671 (21.31)	354 (10.87)	4317 (23.14)	7.58	
Other parties	154 (0.70)	27 (0.83)	127 (0.68)	17.53	
The masses	13912 (63.48)	2250 (69.06)	11662 (62.50)	16.17	
**Education level**					< 0.001
Primary school and below	3412 (15.57)	612 (18.78)	2800 (15.01)	17.94	
Middle school and junior college	8731 (39.84)	1302 (39.96)	7429 (39.82)	14.91	
College degree or above	9773 (44.59)	1344 (41.25)	8429 (45.18)	13.75	
**Marital status**					< 0.001
Never married	8497 (38.77)	807 (24.77)	7690 (41.22)	9.50	
Married	12437 (56.75)	2259 (69.34)	10178 (54.55)	18.16	
Divorce	406 (1.85)	106 (3.25)	300 (1.61)	26.10	
Widowed	576 (2.63)	86 (2.64)	490 (2.63)	14.93	
**Chronic disease**					< 0.001
None	16456 (75.09)	2104 (64.58)	14352 (76.92)	12.79	
One	3512 (16.02)	691 (21.21)	2821 (15.12)	19.68	
More	1948 (8.89)	463 (14.21)	1485 (7.96)	23.77	
**Family income**					0.070
Low	10913 (49.79)	1670 (51.26)	9243 (49.54)	15.30	
High	11003 (50.21)	1588 (48.74)	9415 (50.46)	14.43	
**Residence**					< 0.001
Urban	11811 (53.89)	1631 (50.06)	10180 (54.56)	13.81	
Rural	10105 (46.11)	1627 (49.94)	8478 (45.44)	16.10	
**Depression**					< 0.001
None	9298 (42.43)	1197 (36.74)	8101 (43.42)	12.87	
Mild	7629 (34.81)	1164 (35.73)	6465 (34.65)	15.26	
Moderate	3031 (13.83)	502 (15.41)	2529 (13.55)	16.56	
Severe	1430 (6.52)	269 (8.26)	1161 (6.22)	18.81	
Major	528 (2.41)	126 (3.87)	402 (2.15)	23.86	
**Household type**					< 0.001
Couple Family	3717 (16.96)	607 (18.63)	3110 (16.67)	16.33	
Core family	11574 (52.81)	1366 (41.93)	10208 (54.71)	11.80	
Main family	3836 (17.50)	696 (21.36)	3140 (16.83)	18.14	
Other forms of family	2789 (12.73)	589 (18.08)	2200 (11.79)	21.12	
**Current work status**					< 0.001
Employed	7601 (34.68)	1403 (43.06)	6198 (33.22)	18.46	
Student	6580 (30.02)	433 (13.29)	6147 (32.95)	6.58	
Retirement	2756 (12.58)	493 (15.13)	2263 (12.13)	17.89	
No regular occupation	2609 (11.90)	624 (19.15)	1985 (10.64)	23.92	
Unemployed	2370 (10.81)	305 (9.36)	2065 (11.07)	12.87	
**Negative event**					< 0.001
None	12756 (58.20)	1660 (50.95)	11096 (59.47)	13.01	
One	4945 (22.56)	891 (27.35)	4054 (21.73)	18.02	
More	4215 (19.23)	707 (21.70)	3508 (18.80)	16.77	
**Big five personality**					
Extraversion	6.23 ± 1.62	6.21 ± 1.59	6.24 ± 1.62	-	0.397
Agreeableness	7.00 ± 1.48	6.90 ± 1.49	7.01 ± 1.48	-	< 0.001
Conscientiousness	6.76 ± 1.65	6.79 ± 1.61	6.76 ± 1.66	-	0.237
Nervousness	6.27 ± 1.56	6.43 ± 1.49	6.24 ± 1.56	-	< 0.001
Openness	6.46 ± 1.55	6.25 ± 1.55	6.50 ± 1.55	-	< 0.001

Mean ± standard deviation was used to describe continuous variable, and number (constituent ratio [%]) was used to describe categorical variable

### Family health functioning score

Table 2 shows that the total score of family health scale is 38.65, family social/emotional health processes, family healthy lifestyle, family health resources scores are similar with 12.17, 8.16, 10.54, and family external social supports had the lowest score of 7.79. Non-smokers scored higher than non-smokers on the total family health score as well as on all dimensions.

**Table 2:** Family health and their entry scores for the four dimensions

FHS-SF	Mean ± SD	Currently smoking	
		**Yes**	**No**
**Family Health**	**38.65 ± 0.05**	**37.22 ± 0.13**	**38.90 ± 0.05**
** Family social/emotional health processes**	**12.17 ± 0.02**	**11.66 ± 0.05**	**12.26 ± 0.02**
We support each other	4.06 ± 0.01	3.91 ± 0.02	4.08 ± 0.01
I feel safe in my family relationships	4.04 ± 0.01	3.86 ± 0.02	4.07 ± 0.01
We stay hopeful even in difficult times	4.07 ± 0.01	3.89 ± 0.02	4.10 ± 0.01
**Family healthy lifestyle**	**8.16 ± 0.01**	**7.80 ± 0.04**	**8.22 ± 0.01**
We help each other in seeking health care services when needed (such as making doctor’s appointments)	4.12 ± 0.01	3.95 ± 0.02	4.15 ± 0.01
We help each other make healthy changes	4.03 ± 0.01	3.85 ± 0.02	4.07 ± 0.01
**Family health resources**	**10.54 ± 0.02**	**10.33 ± 0.06**	**10.57 ± 0.02**
We do not trust doctors and other health professionals	3.72 ± 0.01	3.64 ± 0.02	3.73 ± 0.01
My family did not have enough money at the end of the month after bills were paid	3.24 ± 0.01	3.19 ± 0.02	3.25 ± 0.01
My family did not have adequate housing	3.58 ± 0.01	3.49 ± 0.02	3.59 ± 0.01
**Family external social supports**	**7.79 ± 0.01**	**7.43 ± 0.04**	**7.85 ± 0.01**
We have people outside of our family we can turn to when we have problems at school or work	3.90 ± 0.01	3.70 ± 0.02	3.94 ± 0.01
If we needed financial help, we have people outside of our family we could turn to for a loan (e.g., for ¥1000)	3.89 ± 0.01	3.74 ± 0.02	3.91 ± 0.01

The entries of family health resources had been reverse-assigned, with higher scores indicating better

*FHS-SF* Family Health Scale-Short Form, *SD* Standard Deviation

### Gender differences in personality scores

Both female and urban population had higher scores than male and rural population except neurotic personality. In Table 3, the average scores for gender and place of residence are presented for each of the five domains. Independent samples t-tests were used to analyze disparities in gender and place of residence. The analysis of variations in living arrangements was conducted through the use of independent samples t-tests. Table 3 summarizes the effect sizes. In every aspect except neurotic personality, both the female and urban populations outperformed the male and rural populations.

**Table 3:** Mean and SD for Big five personality domains and raw aspect scores

	**Gender**					**Residence**				
	**Females**		**Males**		**t**	**Urban**		**Rural**		**t**
	**Mean**	**SD**	**Mean**	**SD**		**Mean**	**SD**	**Mean**	**SD**	
**Extraversion**	6.277	0.016	6.192	0.015	**-5.767**	6.285	0.015	6.175	0.016	**-7.094**
**Agreeableness**	7.069	0.014	6.923	0.014	-10.468	7.032	0.014	6.954	0.014	-5.425
**Conscientiousness**	6.792	0.016	6.732	0.015	-3.896	6.767	0.015	6.757	0.016	-0.653
**Nervousness**	6.105	0.015	6.437	0.014	**23.427**	6.251	0.015	6.295	0.015	**2.950**
**Openness**	6.542	0.015	6.379	0.014	-11.380	6.645	0.015	6.244	0.015	-27.218

Bolded t values indicate statistically significant effect sizes

### Relationship between FHS and smoking behaviors

Smoking behaviors were significantly associated with FHS according to binary regression models before adjustment(OR = 0.964, *P* < 0.001); after adjusting for age, gender, and household type(adjusted model 1)(OR = 0.972, *P* < 0.001); and after adjusting for above variables, as well as marital, education, income, work status, religion, political, residence, chronic diseases, negative event, depression(adjusted model 2) (OR = 0.974, *P* < 0.001); and after adjustment for the factors in model 2, as well as negative event and depression(adjusted model 3) (OR = 0.981, *P* < 0.001). Smoking behaviors were also significantly correlated with family social/emotional health processes, family healthy lifestyle and family external social supports to binary regression models before and after adjustment (OR = 0.928 ~ 0.948; *P* < 0.001) (OR = 0.891 ~ 0.916; *P* < 0.001) (OR = 0.889 ~ 0.925, *P* < 0.001) (Table 4).

**Table 4:** Binary logistics regression analysis for smoking behaviors associated with family health

Smoking behaviors	Unadjusted		Adjusted 1		Adjusted 2		Adjusted 3	
	**OR (95% CI)**	***P* value**	**OR (95% CI)**	***P* value**	**OR (95% CI)**	***P* value**	**OR (95% CI)**	***P* value**
**Family Health**	0.964 (0.959,0.970)	< 0.001	0.972 (0.966,0.977)	< 0.001	0.974 (0.968,0.980)	< 0.001	0.981 (0.974,0.987)	< 0.001
**Family social/emotional health processes**	0.928 (0.916,0.940)	< 0.001	0.941 (0.928,0.954)	< 0.001	0.935 (0.922, 0.948)	< 0.001	0.948 (0.934,0.962)	< 0.001
**Family healthy lifestyle**	0.891 (0.875,0.908)	< 0.001	0.908 (0.890,0.927)	< 0.001	0.900 (0.881,0.920)	< 0.001	0.916 (0.896,0.937)	< 0.001
**Family health resources**	0.976 (0.965,0.988)	< 0.001	0.997 (0.984,1.009)	0.582	1.007 (0.994,1.020)	0.315	1.021 (1.008,1.036)	0.002
**Family external social supports**	0.889 (0.872,0.906)	< 0.001	0.911 (0.892,0.930)	< 0.001	0.910 (0.890,0.930)	< 0.001	0.925 (0.905,0.946)	< 0.001

Logistic regression model was applied measure the association between family health (independent variable) and smoking behaviors (dependent variable)

Adjusted 1: Adjusting for age, gender and household type

Adjusted 2: Adjusting for age, gender, household type, marital status, education levels, family income, current work status, religion, political landscape, residence, chronic diseases

Adjusted 3: Adjusting for age, gender, household type, marital, education, income, work status, religion, political, residence, chronic diseases, negative event, depression

*P*-value less than 0.001 was considered conservative for statistical significance after Bonferroni correction

*OR* Odds ration, *CI* confidence interval

### Relationship between smoking behaviors and Big Five personality

Extraversion, nervousness, and openness were significantly associated with smoking after adjusting all variables (OR = 0.970 ~ 1.281, *P* < 0.001). Agreeableness and conscientiousness were not significantly related to smoking after adjusting all variables (*P* > 0.001) (Table 5).

**Table 5:** Binary logistics regression analysis for smoking behaviors associated with Big Five personality

Smoking behaviors	**Unadjusted**		**Adjusted 1**		**Adjusted 2**		**Adjusted 3**	
	**OR (95%CI)**	***P* value**	**OR (95%CI)**	***P* value**	**OR (95%CI)**	***P* value**	**OR (95%CI)**	***P* value**
**Big Five personality**								
**Extraversion**	0.990(0.968, 1.013)	0.397	1.015(0.990,1.041)	0.240	1.027(1.001,1.053)	0.046	1.048(1.021,1.075)	< 0.001
**Agreeableness**	0.952(0.928,0.976)	< 0.001	0.982(0.955,1.009)	0.184	0.973(0.947,1.001)	0.055	1.000(0.972,1.029)	0.973
**Conscientiousness**	1.014(0.991,1.037)	0.237	1.015(0.991,1.041)	0.223	0.981(0.956,1.006)	0.129	1.020(0.993,1.047)	0.145
**Nervousness**	1.079(1.054,1.106)	< 0.001	1.014(0.987,1.041)	0.319	1.008(0.982,1.136)	0.542	1.063(1.033,1.093)	< 0.001
**Openness**	0.901(0.880,0.924)	< 0.001	0.940(0.915,0.965)	< 0.001	0.974 (0.948,1.001)	0.060	0.970(0.943,0.997)	0.029

Logistic regression model was applied measure the association between Big Five personality (independent variable) and smoking behaviors (dependent variable)

Adjusted 1: Adjusting for age, gender and household type

Adjusted 2: Adjusting for age, gender, household type, marital status, education levels, family income, current work status, religion, political landscape, residence, chronic diseases

Adjusted 3: Adjusting for age, gender, household type, marital, education, income, work status, religion, political, residence, chronic diseases, negative event, depression

*P*-value less than 0.001 was considered conservative for statistical significance after Bonferroni correction

*OR* Odds ration, *CI* confidence interval, *FHS* family health scale

### Relationship between FHS and Big Five personality

FHS was significantly associated with all of the personalities according to the linear regression models before adjustment (β = 0.032 ~ 0.074, *P* < 0.001); after adjusting for age, gender, and household type(adjusted model 1)(β = 0.030 ~ 0.073, *P* < 0.001); and after adjusting for above variables, as well as marital, education, income, work status, religion, political, residence, chronic diseases, negative event, depression(adjusted model 2) (β = 0.026 ~ 0.073, *P* < 0.001); and after adjustment for the factors in model 2, as well as negative event and depression(adjusted model 3) (β = 0.026 ~ 0.064, *P* < 0.001) (Table 6).

**Table 6:** Liner regression analysis for Big Five personality associated with family health

Big Five personality	Unadjusted		Adjusted 1		Adjusted 2		Adjusted 3	
	**β(95%CI)**	***P* value**	**β(95%CI)**	***P* value**	**β(95%CI)**	***P* value**	**β(95%CI)**	***P* value**
**Extraversion**	0.038(0.035,0.041)	< 0.001	0.037(0.034,0.041)	< 0.001	0.036(0.033,0.040)	< 0.001	0.028(0.025,0.032)	< 0.001
**Agreeableness**	0.074(0.071,0.077)	< 0.001	0.073(0.070,0.076)	< 0.001	0.073(0.071,0.076)	< 0.001	0.064(0.061,0.067)	< 0.001
**Conscientiousness**	0.063(0.060,0.067)	< 0.001	0.064(0.061,0.067)	< 0.001	0.064(0.061,0.067)	< 0.001	0.048(0.045,0.051)	< 0.001
**Nervousness**	0.042(0.039,0.045)	< 0.001	0.045(0.042,0.048)	< 0.001	0.047(0.044,0.050)	< 0.001	0.027(0.024,0.031)	< 0.001
**Openness**	0.032(0.029,0.035)	< 0.001	0.030(0.027,0.033)	< 0.001	0.026(0.023,0.029)	< 0.001	0.026(0.023,0.029)	< 0.001

Logistic regression model was applied to measure the association between family health (independent variable) and Big Five personality (dependent variable)

Adjusted 1: Adjusting for age, gender, and household type

Adjusted 2: Adjusting for age, gender, household type, marital status, education levels, family income, current work status, religion, political landscape, residence, and chronic diseases

Adjusted 3: Adjusting for age, gender, household type, marital, education, income, work status, religion, political, residence, chronic diseases, negative event, depression

*P*-value less than 0.001 was considered conservative for statistical significance after the Bonferroni correction

*β* Beta, *CI* confidence interval

### Mediating effect of Big five personality on the relationship between FHS and smoking

**Fig. 1:**
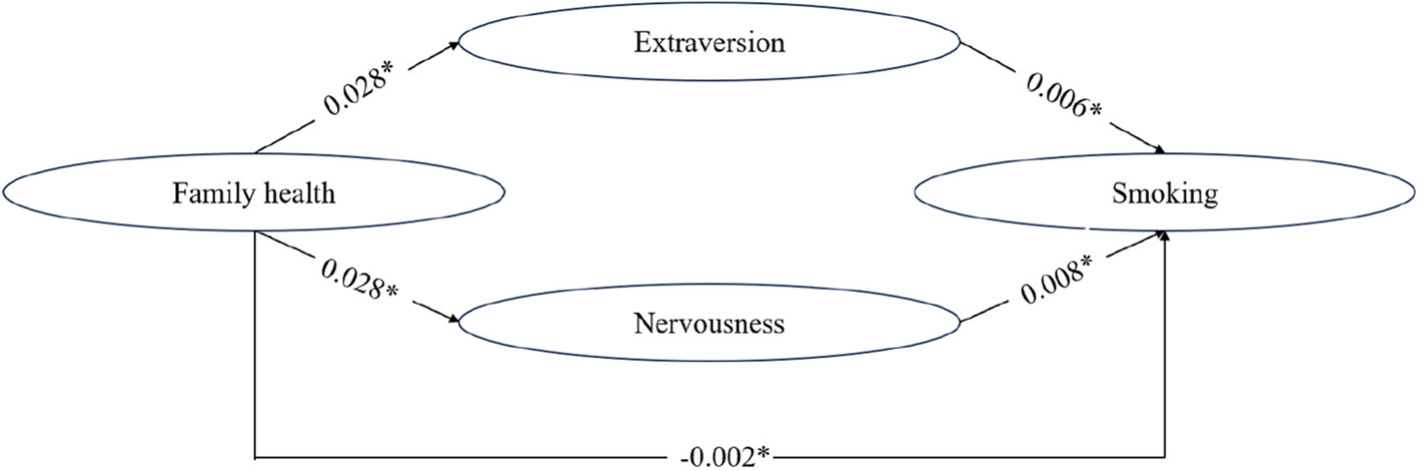
Mediation analysis. The Sobel test was used to test the hypothesis that the indirect role was equal to 0, adjusting for potential confounders (age, gender, and household type, marital, education, income, work status, religion, political, residence, chronic diseases, negative event, depression). Values are bolded if they achieved statistical significance at *p* ≤ 0.001

Through mediation analysis, it was discovered that family health had a negative impact of -0.002 on smoking. The relationship between family health and smoking was influenced by extraversion and nervousness. The results of the Sobel test were statistically significant (*P* < 0.001). The analysis of KHB composition indicated that the two agents decreased the overall impact of family health on smoking by 17.00%. Furthermore, the moderating impacts of extroversion and anxiety accounted for 47.81% and 52.19%, correspondingly, surpassing the remaining three mediators significantly. Additional information was provided in Table 7 and Fig. 1. We explored the mediating effects of big five personality on the 4 dimensions of family health, the results were similar (Table 8).

**Table 7:** The mediating effect of Big Five personality on family health and smoking behaviors explored by Sobel tests and Karlson-Holm-Breen (KHB) decomposition methods

	**Extraversion**	**Agreeableness**	**Conscientiousness**	**Nervousness**	**Openness**
Family health → mediator coefficient	0.028^*^	0.064^*^	0.049^*^	0.028^*^	0.026^*^
Mediator → smoking coefficient	0.006^*^	0.003	0.003	0.008^*^	-0.002
Indirect effect, β	0.001^*^	0.001^*^	0.001	0.001^*^	-0.000
Direct effect, β	-0.003^*^	-0.003^*^	-0.003^*^	-0.003^*^	-0.002^*^
Total effect, β	-0.002^*^	-0.002^*^	-0.002^*^	-0.002^*^	-0.002^*^
Proportion of total effect that is mediated	-0.066	-0.094	-0.065	-0.092	0.019
Sobel test	3.821^*^	2.125	2.074	4.848^*^	-1.116
**KHB decomposition**					
Proportion of mediation effect (%)	47.81	--^a^	--	52.19	--

*P*-value less than 0.001 was considered conservative for statistical significance after Bonferroni correction

^*^p < 0.001

^a^Agreeableness, conscientiousness and openness were not taken into KHB analysis as the mediation effects were not significant

**Table 8:** Mediation analysis and Karlson-Holm-Breen (KHB) decomposition of Big Five personality mediating the associations between dimensions of family health and smoking behaviors

	**Extraversion**	**Agreeableness**	**Conscientiousness**	**Nervousness**	**Openness**
**Family social/emotional health processes**					
Family health → mediator coefficient	0.375^*^	0.272^*^	0.145^*^	0.118^*^	0.353^*^
Mediator → smoking coefficient	0.006^*^	-0.001	0.001	0.006^*^	-0.007
Indirect effect, β	0.002^*^	-0.000	0.000	0.001^*^	-0.002
Direct effect, β	-0.002	0.001	0.001	-0.000	0.003
Total effect, β	0.001	0.001	0.001	0.001	0.001
Sobel test	3.433^*^	-0.379	0.456	4.066^*^	-3.322
**KHB decomposition**					
Proportion of mediation effect (%)	79.33	--	--	20.67	--
**Family healthy lifestyle**					
Family health → mediator coefficient	0.155^*^	0.198^*^	0.519^*^	0.481^*^	0.056^*^
Mediator → smoking coefficient	0.004	-0.001	-0.006	0.006	-0.003
Indirect effect, β	0.001	-0.000	-0.003	0.003	-0.000
Direct effect, β	0.003	0.003^*^	0.006^*^	0.000	0.003
Total effect, β	0.003	0.003	0.003	0.003	0.003
Sobel test	2.507	-0.881	-2.752	2.752	-2.112
**KHB decomposition**					
Proportion of mediation effect (%)	--	--	--	--	--
**Family health resources**					
Family health → mediator coefficient	0.123^*^	0.119^*^	0.173^*^	0.128^*^	0.077^*^
Mediator → smoking coefficient	0.001	-0.004	-0.004	0.003^*^	-0.005
Indirect effect, β	0.000	-0.000	-0.001	0.000	-0.000
Direct effect, β	0.012^*^	0.012^*^	0.013^*^	0.012^*^	0.012^*^
Total effect, β	0.012^*^	0.012^*^	0.012^*^	0.012^*^	0.012^*^
Sobel test	0.876	-2.361	-2.767	1.644	-3.377
**KHB decomposition**					
Proportion of mediation effect (%)	--	--	--	--	--
**Family external social supports**					
Family health → mediator coefficient	0.064^*^	0.086^*^	0.078^*^	0.051^*^	0.037^*^
Mediator → smoking coefficient	0.006^*^	0.004	0.004	0.008^*^	-0.002
Indirect effect, β	0.000^*^	0.000	0.000	0.000^*^	-0.000
Direct effect, β	-0.006^*^	-0.006^*^	-0.006^*^	-0.006^*^	-0.006^*^
Total effect, β	-0.006^*^	-0.006^*^	-0.006^*^	-0.006^*^	-0.006^*^
Sobel test	4.460^*^	2.213	2.409	5.214^*^	-1.035
**KHB decomposition**					
Proportion of mediation effect (%)	56.95	--	--	43.05	--

The Sobel test was used to test the hypothesis that the indirect role was equal to 0, adjusting for potential confounders (age, gender, household type, marital, education, income, work status, religion, political, residence, chronic diseases, negative event, depression). Values are marked with *  if they achieved statistical significance at *p* ≤ 0.001

### Subgroup analysis

Table 9 displays the subgroup analysis of sex and place of residence. The connection between FHS scores and smoking in females and rural people was partially influenced by extraversion and nervousness (z = 3.960; indirect effect = 0.000, *P* < 0.001; direct effect = -0.002, *P* < 0.001) (z = 4.205;indirect effect = 0.000, *P* < 0.001; direct effect = -0.002, *P* < 0.001)and (z = 3.429; indirect effect = 0.000, *P* < 0.001; direct effect = -0.002, *P* < 0.001) (z = 4.149;indirect effect = 0.000, *P* < 0.001; direct effect = -0.002, *P* < 0.001).

**Table 9:** Subgroup mediation analysis stratified by gender and residence based on Sobel tests and Karlson-Holm-Breen (KHB) decomposition methods

	**Extraversion**	**Agreeableness**	**Conscientiousness**	**Nervousness**	**Openness**
**Male**					
Family health → mediator coefficient	0.026^*^	0.063^*^	0.049^*^	0.039^*^	0.026^*^
Mediator → smoking coefficient	0.011^*^	0.010^*^	0.010^*^	0.013^*^	-0.006^*^
Indirect effect, β	0.000	0.001^*^	0.000	0.000^*^	-0.000
Direct effect, β	-0.002^*^	-0.003^*^	-0.002^*^	-0.002^*^	-0.002
Total effect, β	-0.002	-0.002	-0.002^*^	-0.002	-0.002
Sobel test	3.960^*^	3.196	3.397	4.205^*^	-2.205
**KHB decomposition**					
Proportion of mediation effect (%)	43.48	--	--	56.52	--
**Female**					
Family health → mediator coefficient	0.031^*^	0.064^*^	0.050^*^	0.016^*^	0.027^*^
Mediator → smoking coefficient	0.002	-0.002	-0.002	0.004^*^	0.002
Indirect effect, β	0.000	-0.000	0.000	0.000	0.000
Direct effect, β	-0.003^*^	-0.003^*^	-0.003^*^	-0.003^*^	-0.003^*^
Total effect, β	-0.003^*^	-0.003^*^	-0.003^*^	-0.003^*^	-0.003^*^
Sobel test	1.491	-1.979	-1.651	2.972	1.971
**KHB decomposition**					
Proportion of mediation effect (%)	--	--	--	--	--
**Rural**					
Family health → mediator coefficient	0.028^*^	0.057^*^	0.041^*^	0.025^*^	0.035^*^
Mediator → smoking coefficient	0.007^*^	0.004	0.004	0.009^*^	-0.001
Indirect effect, β	0.000^*^	0.000	0.000	0.000^*^	-0.000
Direct effect, β	-0.002^*^	-0.002^*^	-0.002^*^	-0.002^*^	-0.001
Total effect, β	-0.001^*^	-0.001^*^	-0.001^*^	-0.001^*^	-0.001^*^
Sobel test	3.429^*^	1.961	2.026	4.149^*^	-0.378
**KHB decomposition**					
Proportion of mediation effect (%)	52.36	--	--	47.64	--
**Urban**					
Family health → mediator coefficient	0.028^*^	0.073^*^	0.060^*^	0.032^*^	0.015^*^
Mediator → smoking coefficient	0.005	0.003	0.002	0.006	-0.003
Indirect effect, β	0.000	0.000	0.000	0.000	-0.000
Direct effect, β	-0.004^*^	-0.004^*^	-0.004^*^	-0.004^*^	-0.003^*^
Total effect, β	-0.003^*^	-0.003^*^	-0.003^*^	-0.003^*^	-0.003^*^
Sobel test	2.041	1.113	0.823	2.620	-1.329
**KHB decomposition**					
Proportion of mediation effect (%)	--	--	--	--	--

The Sobel test was used to test the hypothesis that the indirect role was equal to 0, adjusting for potential confounders (age, gender, household type, marital, education, income, work status, religion, political, residence, chronic diseases, negative event and depression). Values are marked with * if they achieved statistical significance at *p* ≤ 0.001

## Discussion

### Hypothesis 1 Family health is associated with smoking behavior

The present study found that higher total family health scores were negatively associated with smoking behavior. Previous research has demonstrated that both internal and external family support can assist in smoking cessation, such as encouragement and guidance from family members, help from health professionals, and community culture [46], which can promote smoking cessation among smokers. Specifically, better family social/emotional health processes, family healthy lifestyle, and family external social support scores were associated with reduced smoking behavior.A healthy family lifestyle may serve as a role model for healthy behaviors to reduce smoking behaviors, while the emotional connection and emotional support provided by the family is conducive to helping individuals establish good mental health through sound family functioning, and is more likely to avoid unhealthy behaviors such as smoking in socialization behaviors [3]. Some studies have shown that external social support from the family may convey positive social expectations based on external support from a wider social network of community and friends, and that social support can provide practical help such as financial assistance, employment guidance, and psychological assistance [49, 50] to make them more capable of coping with challenges in life and less likely to cope with stress through smoking. In addition, the unadjusted regression model showed that good family health resource scores were negatively associated with smoking behavior, while the final adjusted regression model showed a positive association. This may be due to the fact that the family health resources dimension includes entries measuring the family’s economic resources, and there is a complex interaction effect between economic resources and smoking behavior that affects the stability of the results. In one case, smoking may be a coping mechanism for people living in poverty [10]. In the other case, a better economic standard of living provides the conditions for individuals to purchase tobacco products on the premise that basic material needs can be met. When faced with economic pressures, non-basic necessities such as cigarettes may become burdensome, leading individuals to quit smoking [48]. Future research needs to delve further into the mechanisms underlying this relationship and consider additional covariates and potential confounders to better understand the process of shaping and maintaining smoking behavior. We also note that the mediating effect in this study was small. Family health may provide a critical research perspective not clinically, but socially, because family health encompasses a wealth of information and the family is a very important partner in the delivery of many community health services. We have initially explored the mechanisms by which family health influences individual health behaviors, and the mechanisms do exist. In addition, the mechanisms by which family health acts on health behaviors may be complex, and some mediating effects may be overshadowed by direct effects. We can develop family health promotion programs and community health education programs, which will lead to more effective prevention and control of smoking behaviors and promotion of the public’s physical and mental health.

### Hypothesis 2 Personality traits are associated with family health and smoking behavior, respectively, and mediate the association between family health and smoking behavior

The present study found that higher levels of extraversion, agreeableness, conscientiousness, nervousness, openness were all associated with higher levels of family health. Previous studies have suggested that family health is an important objective situational factor that influences personality formation [22]. Family systems theory proposes that an individual’s family-of-origin environment has a direct relationship to emotional expression, behavioral patterns, and intimate relationships, which significantly impact an individual’s physical and mental health and can be used to predict psychological problems [32].

The findings also suggest that high extraversion is a risk factor for smoking and may be related to the smoking-related polymorphism in the BDNF gene rs6265 [35]. Highly extraverted smokers tend to conform to in-group norms, leading to tobacco dependence. Additionally, highly extraverted individuals are more likely to be identified with habitual behaviors due to their lively, outgoing, and expressive styles, increasing the number of observed smoking episodes [25]. High nervousness is also a risk factor for smoking and may be associated with a greater likelihood of facing problems with negative emotions (e.g., anxiety, fear, irritability, frustration, and depression) [17]. Because tobacco-induced 5-hydroxytryptophan suppresses negative emotions [31], people who are high in nervousness are at higher risk of smoking and relapse. Unlike previous studies [52], the present study found high openness to be a protective factor for smoking. On the one hand, high openness tends to participate in a wide range of activities in daily life, have a wide range of interests, and pay more attention to things other than smoking [40]. On the other hand, high openness is more likely to be exploratory in its motivation to smoke and is also more capable of dealing with withdrawal symptoms and more likely to stop smoking [52]. The reasons why pleasantness and responsibility were not associated with smoking behavior in this study require further analysis. It may indicate that nicotine dependence and sociodemographic characteristics outweigh the influence of personality on smoking behavior.

The results of the mediation analyses suggest that extraversion and nervousness play a partial mediating role between family health and smoking. The results of the mediation analyses suggest that extraversion and nervousness play a partial mediating role between family health and smoking. Family health both directly related to smoking and indirectly related to smoking through extraversion and nervousness personalities, overall weakening the protective effect of family health on smoking. This finding will need to be verified by adding longitudinal studies in the future. Better levels of family health may provide higher levels of emotional support for personality formation, such as emotionally warm parenting, which is associated with the formation of extraversion [2]. Good levels of family health also contribute to the creation of agreeableness and positive emotions, which have been shown to be strongly associated with high extraversion [1]. However, as mentioned earlier, high extraversion is associated with smoking behavior, and people with higher extraversion are more accepting of smoking [5]. Therefore, high extraversion counteracts the link between family health and smoking. The family environmental context of high neurotic personality formation is still understudied, and our finding that higher levels of family health are associated with the formation of high neurotic personality seems to contradict previous studies [24] suggesting that a family climate with high levels of family conflict, disconnection between parents, and so on, contributes to the development of a high neurotic personality. This discrepancy may be due to the different conceptualizations and measurements of family health in our study. Family health is viewed as a comprehensive concept rather than a specific event [27]. The current research additionally validated that elevated nervousness is closely linked to smoking behavior [11], consequently, heightened anxiety also undermines the connection between family health and smoking.

In the gender subgroups, personality mediated the effect of family health on smoking in males, while the relationship between personality and smoking behavior was not significant in females. Our study confirmed the findings of previous research that indicate gender disparities in personality traits. Specifically, females scored higher than males in all four personality dimensions, except for nervousness where males scored lower. Differences in gender norms, as well as variances in evolutionary attention to offspring investment across sexes in biology and evolutionary theory, could potentially explain the disparities in personality between males and females [44]. Gender differences in mediated pathways may be related to the social normative influence of cigarette smoking culture [13], to the fact that Chinese women’s smoking rates are much lower than men’s, and to the fact that there were very few female smokers in this study, making personality reveal a limited influence on smoking. Second, women reported more positive and negative emotional events [6], which implies that women’s behavioral mechanisms may be more complex, and that there may be multiple pathways of influence on smoking, thus masking the influence of personality. In the residence subgroup, personality mediated the effect of family health on smoking among rural residents, consistent with results from the total population, yet this pathway was not significant among urban residents. Significant differences exist between urban and rural areas in China in terms of social environment, education level, cultural background, and economic status [18]. The study showed that rural residents had higher mean scores on all five personality traits than urban residents. The possible reason for this is that urban residents live in a more diverse environment with more complex community networks, information and challenges, and richer and more multidimensional resources and support available [30], which somewhat weakens the influence of personality traits on their behavior. At the same time, the generally better economic situation and education level in the city also help individuals to better understand their own behavior and that of others [33], which helps them to overcome the influence of personality traits to some extent.

In summary, this study reveals that extraversion and neuroticism weaken the protective effect of family health on smoking. Therefore, to increase smoking cessation rates, we should improve family health, especially in the dimensions of family health resources and family external social supports. At the level of family health resources, families should be guided to make rational use of health resources, increase spending on family health investments such as insurance, medical checkups, and physical fitness for family members [16], and reduce purchases of non-essential consumer goods such as tobacco use. Research has demonstrated that within the realm of family social support, cravings associated with smoking are typically triggered by external cues, such as physically holding a cigarette [4]. Additionally, it has been found that behavior can be influenced by both descriptive social norms (observed actions of others) and injunctive norms (others’ expectations of what one should do) in separate ways [14]. Hence, in terms of societal assistance beyond one’s residence, it is imperative to enforce laws that promote homes free from smoking in order to decrease the presence of smoking triggers in a person’s surroundings. Additionally, it is crucial to consistently monitor smoking habits in behavioral treatments (for instance, by reducing the amount of cigarettes consumed or the frequency of cravings), thus enhancing the rate of quitting smoking among individuals with mental health issues. Alternatively, focusing on personality as a mediator between family health and smoking could be a potential approach for interventions aimed at quitting smoking. For highly neurotic groups, a healthier social identity could be developed to reduce anxiety-induced smoking by enriching positive social networks and increasing meaningful activities [12]. For highly extraverted individuals, it is possible to help them recognize possible dependence on the external environment and reduce the impact of social group smoking on individual behavior by enhancing individual self-concept [23]. Personality is both stable and malleable, and the formation of new behavioral traits and thought patterns through individual cognitive regulation and practical development can help to enhance the protective impact of family health on smoking. While both men and rural groups may be able to improve smoking through family health and personality, there may be more complex pathways of influence for women and urban residents that need to be explored in the future.

#### Implications for policy and practice

For individuals with high extroversion scores, social support can be provided to help them reduce their dependence on smoking by enhancing their social support and satisfaction through participation in more social and group activities such as sports, cultural activities, and community service. For individuals with higher extroversion scores, policies can reduce their chances of smoking by restricting smoking behaviors in social situations, such as implementing stricter smoking bans in public places. For individuals with high stress scores, provide government-purchased and subsidized outreach mental health education and counseling services, as well as psychological interventions such as cognitive-behavioral therapy, relaxation training, and meditation, in order to reduce the need for those with high stress scores to smoke to relieve stress and anxiety.

Public tobacco control policies should fully consider family factors, design and implement family-specific tobacco control intervention programs, and reduce the incidence of smoking behaviors by upgrading family health education and improving the family environment through health education activities and mental health support. Secondly, family health promotion programs can be developed. These programs may include family communication skills training, healthy lifestyle education, and mental health seminars, aiming to enhance family members’ awareness of the harms of smoking, improve their health literacy and mental health, and educate family members about the harms of smoking and how to establish a healthy lifestyle. In addition, the policy should encourage and support mutual monitoring and support among family members in order to enhance the role of the family in the process of tobacco control. At the same time, family health resources and external family support should be strengthened to include family health as a practice strategy or assessment indicator for health promotion. This applies particularly to community/family/geriatric health care providers, who need interdisciplinary thinking to provide home-based tobacco cessation services and guidance. This can be done through health education activities such as community health education programs, tobacco control awareness campaigns, and volunteer trainings to create a positive and healthy community environment. Finally, community healthcare organizations should enhance assessment and intervention in clinical practice. In clinical practice, physicians and other healthcare providers should assess patients’ smoking status and provide individualized advice and support to quit. This can include a variety of approaches such as medication, psychotherapy, and behavioral therapy. Healthcare providers can offer resources such as smoking cessation programs and group therapy to help patients quit smoking and maintain long-term abstinence.

## Limitations

This study has various restrictions. Initially, our investigation relied on cross-sectional data, and the results can only illustrate associations and do not establish causality. The mechanisms underlying the effects of family health and Big five personality on smoking behavior are complex and need to be further explored in the future through prospective analyses. Secondly, data on smoking, depression, personality, and FHS were obtained using self-administered questionnaire measures, and the results may be affected by self-report bias and social desirability bias. In addition, negative emotions mainly involved studies on depression, anxiety, and perceived stress, and there was a lack of exploration of other negative emotions such as anger, loneliness, and panic. Future studies should focus on the inclusion of these emotions. Meanwhile, although we adjusted for control variables in our study, it is possible that there are unmeasured confounders (e.g., lifestyle, demographic variables, etc.) that are subject to omitted variable bias. This leads to the possibility that an individual’s unobserved effect may be correlated with an observed variable or that the estimated effect includes the role of these unobserved factors. Finally, the effect coefficients in the mediator analysis of the relationship between family health and smoking as influenced by extroversion and tension were small, and further exploration needs to be made in the future as to how family health actually influences individual health behavior. Finally, the effect coefficients in the mediator analysis of the relationship between family health and smoking as influenced by extroversion and tension were small, and further exploration needs to be made in the future as to how family health actually influences individual health behavior.

## Conclusions and implications

In this large national population-based cross-sectional study, we examined differences between smokers and nonsmokers in the study population through descriptive statistics and explored the associations between family health, Big five personality, and smoking behavior, as well as the mediating role of Big five personality between family health and smoking behavior. Our findings suggest that family health and personality may be important factors to consider in smoking cessation interventions aimed at improving smoking outcomes. Based on our results, interventions that target diverse personality traits could prove effective in promoting healthy behaviors among Chinese residents. Our study provides a foundation for developing an intervention strategy for smoking cessation programs that focus on the interaction between family health and personality diversity.

### Supplementary Information

**Supplementary Material 1.**

## Data Availability

The datasets generated and/or analyzed during the current study are not publicly available due to limitations of ethical approval involving patient data and anonymity but are available from the corresponding author upon reasonable request.
