# Supplementary material for: The association between smoking and family health with the mediation role of personality among Chinese people: nationwide cross-sectional study
Source: BMC Psychiatry. 2024 Mar 14;24:206. doi: 10.1186/s12888-024-05654-x (PMC10941408; doi:10.1186/s12888-024-05654-x)
Supplement: Supplementary file 1 — Supplementary Material 1. [file 12888_2024_5654_MOESM1_ESM.docx]

Supplementary Material

**Supplementary Table 1** Variable description.

**Supplementary Table 2** The Selection Process of Covariates: Step 1 - Analyzing the Relationship Between the Covariate and Y (Y=Currently smoking) One by One.

**Supplementary Table 3** The Selection Process of Covariates: Step 2 - Covariates Were Introduced into The Basic Model and Removed from The Complete Model to Observe the Change of the Regression Coefficient of X (X=Family health, Family social/emotional health processes, Family healthy lifestyle, Family health resources, Family external social supports).

**Supplementary Table 4** The Selection Process of Covariates: Step 2 - Covariates Were Introduced into The Basic Model and Removed from The Complete Model to Observe the Change of the Regression Coefficient of X (X=extraversion, agreeableness, conscientiousness, nervousness, openness).Supplementary Table 1 Variable description.

| **Variable name** | **Definition or code** |
| --- | --- |
| **Dependent variable** |  |
| Smoking | It indicates the respondent’s current smoking habit.0=None; 1=Yes |
| **Mediation variable** |  |
| Big five personality | It was measured using the Big Five Classic Inventory of Personality (BFI-10), which consists of 10 items with 5 dimensions, using a 5-point Likert scale. The five dimensions were extraversion, agreeableness, conscientiousness, neuroticism, and openness. The total scores of each dimensions form 2-10, higher scores predict higher preference of the personality. |
| **Independent** |  |
| Family health | The family health status was measure via Family Health Scale (Short-Form) which consists of a total of 10 items, each item is rated on a five-point Richter scale. Questions 1, 2, 3, 4, 5, 7 and 8 are positively scored: Strongly disagree =1; Somewhat disagree =2; Neither agree nor disagree =3; Somewhat agree =4; Strongly agree =5; Questions 6, 9 and 10 are negative scored: Strongly disagree =5; Somewhat disagree =4; Neither agree nor disagree =3; Somewhat agree =2; Strongly agree =1. The total score is the sum of each question, with a minimum score of 10 and a maximum score of 50, the higher the score, the higher the score, the better the family health index. |
| **Control variables** |  |
| Age stage | 1=12-17 years; 2=18-59 years; 3= ≥60 years |
| Gender | 1=Male; 2=Female. |
| Religion | 0=None; 1=Yes. |
| Political landscape | 1=Party member or Probationary Party; 2=Member of the Communist Youth League; 3=Other parties; 4=The masses |
| Education levels | Education levels is highest level of education. 1=Primary school and below; 2=Middle school and junior college; 3=College degree or above |
| Marital status | 1=Never Married; 2=Married, including the first marriage with a spouse, and then remarried with a spouse; 3=Divorce; 4=Widowed. |
| Chronic disease | Chronic diseases diagnosed by doctors 0=No chronic disease; 1=Suffer from a chronic disease; 2=Suffering from various chronic diseases |
| Family income | Family income is the respondent's current monthly per capita family income, which is calculated as total household income divided by the total number of people, where 1=Low (less than $3,000), 2=Moderate (between $3,001 and $5,000), and 3=High (more than $5,001). |
| Registered permanent residence | 1=Urban; 2=Rural |
| Current work status | 1=Employed; 2=Student;3=Retirement; 4=No regular occupation; 5=Unemployed |
| Household type | 1=Couple Family; 2=Core family; 3=Main family; 4=Other forms of family |
| Negative event | 0=None; 1=One; 2=More |
| Depression | Measure with Patient Health Questionnaire; 0=None; 1=Mild; 2=Moderate; 3=Severe; 4=Major |

# Supplementary Table 2 The Selection Process of Covariates: Step 1 - Analyzing the Relationship Between the Covariate and Y (Y=Currently smoking) One by One.

| Covariates | term | beta | Se. | 95CI Low | 95CI Up | *P*-value |
| --- | --- | --- | --- | --- | --- | --- |
| Religion | None | 0.3530 | 0.1011 | 1.1674 | 1.7352 | 0.0005 |
| Political landscape | Member of the Communist Youth League | -0.8445 | 0.0749 | 0.3711 | 0.4978 | <0.0001 |
|  | Other parties | 0.0814 | 0.2320 | 0.6885 | 1.7094 | 0.7257 |
|  | The masses | 0.0313 | 0.0536 | 0.9289 | 1.1461 | 0.5591 |
| Current work status | Student | -1.0989 | 0.0664 | 0.2926 | 0.3796 | <0.0001 |
|  | Retirement | 0.2103 | 0.0895 | 1.0354 | 1.4707 | 0.0188 |
|  | No regular occupation | 0.4113 | 0.0602 | 1.3408 | 1.6978 | <0.0001 |
|  | Unemployed | -0.1595 | 0.0822 | 0.7257 | 1.0016 | 0.0523 |
| Education level | Middle school and junior college | -0.3432 | 0.0622 | 0.6280 | 0.8015 | <0.0001 |
|  | College degree or above | -0.6195 | 0.0656 | 0.4733 | 0.6120 | <0.0001 |
| Chronic disease | None | 0.4548 | 0.0533 | 1.4196 | 1.7492 | <0.0001 |
|  | One | 0.7206 | 0.0672 | 1.8020 | 2.3449 | <0.0001 |
| Household type | Core family | -0.2198 | 0.0577 | 0.7169 | 0.8988 | 0.0001 |
|  | Main family | 0.1749 | 0.0659 | 1.0467 | 1.3555 | 0.0080 |
|  | Other forms of family | 0.4928 | 0.0691 | 1.4294 | 1.8744 | <0.0001 |
| Marital status | Married | 0.7565 | 0.0505 | 1.9298 | 2.3526 | <0.0001 |
|  | Divorce | 1.3295 | 0.1330 | 2.9116 | 4.9049 | <0.0001 |
|  | Widowed | 0.8663 | 0.1404 | 1.8058 | 3.1315 | <0.0001 |
| Family income | High | -0.1738 | 0.0409 | 0.7758 | 0.9105 | <0.0001 |
| Negative event | None | 0.4656 | 0.0489 | 1.4474 | 1.7533 | <0.0001 |
|  | One | 0.4746 | 0.0535 | 1.4473 | 1.7849 | <0.0001 |
| Depression | None | 0.3099 | 0.0473 | 1.2427 | 1.4956 | <0.0001 |
|  | Mild | 0.3683 | 0.0617 | 1.2806 | 1.6312 | <0.0001 |
|  | Moderate | 0.4603 | 0.0796 | 1.3556 | 1.8521 | <0.0001 |
|  | Severe | 0.8804 | 0.1172 | 1.9168 | 3.0346 | <0.0001 |
| Registered permanent residence | Rural | 0.2454 | 0.0410 | 1.1794 | 1.3852 | <0.0001 |

# Supplementary Table 3 The Selection Process of Covariates: Step 2 - Covariates Were Introduced into The Basic Model and Removed from The Complete Model to Observe the Change of the Regression Coefficient of X (X=Family health, Family social/emotional health processes, Family healthy lifestyle, Family health resources, Family external social supports).

| Covariates | Basic model | | | | | Complete model | | | | | Selected |
| --- | --- | --- | --- | --- | --- | --- | --- | --- | --- | --- | --- |
|  | FHS | Family social/emotional health processes | Family healthy lifestyle  processes | Family health resources | Family external social supports | FHS | Family social/emotional health processes | Family healthy lifestyle  processes | Family health resources | Family external social supports |  |
| Original coefficient | -0.0313 | -0.0210 | 0.0081 | 0.1081 | -0.0566 | -0.0214 | 0.0153 | 0.0135 | 0.1077 | -0.0503 |  |
| Religion | -0.0309 | -0.0210 | 0.0085 | 0.1080 | -0.0565 | -0.0214 | 0.0154 | 0.0135 | 0.1078 | -0.0503 |  |
| Political landscape | -0.0310 | -0.0139 * | -0.0097 * | 0.1083 | -0.0565 | -0.0214 | 0.0149 | 0.0141 | 0.1073 | -0.0504 | Yes |
| Current work status | -0.0308 | -0.0089 * | -0.0258 * | 0.1062 | -0.0567 | -0.0224 | 0.0129 * | 0.0188 * | 0.1080 | -0.0512 | Yes |
| Education level | -0.0303 | -0.0145 * | 0.0048 * | 0.1137 | -0.0557 | -0.0218 | 0.0125 * | 0.0133 | 0.1055 | -0.0508 | Yes |
| Chronic disease | -0.0321 | -0.0185 * | 0.0073 | 0.1064 | -0.0554 | -0.0203 | 0.0154 | 0.0148 | 0.1072 | -0.0505 | Yes |
| Household type | -0.0275 * | -0.0168 * | 0.0128 * | 0.1105 | -0.0524 | -0.0227 | 0.0147 | 0.0129 | 0.1055 | -0.0520 | Yes |
| Marital status | -0.0330 | -0.0126 * | -0.0209 * | 0.1062 | -0.0580 | -0.0208 | 0.0148 | 0.0159 * | 0.1089 | -0.0496 | Yes |
| Family income | -0.0306 | -0.0190 | 0.0072 * | 0.1101 | -0.0559 | -0.0215 | 0.0149 | 0.0138 | 0.1073 | -0.0505 | Yes |
| Negative event | -0.0298 | -0.0211 | 0.0228 * | 0.1093 | -0.0537 | -0.0213 | 0.0178 * | 0.0091 * | 0.1035 | -0.0525 | Yes |
| Depression | -0.0252 * | -0.0061 * | 0.0463 * | 0.1001 | -0.0567 | -0.0260 * | 0.0012 * | -0.0118 * | 0.1138 | -0.0481 | Yes |
| Registered permanent residence | -0.0297 | -0.0176 * | 0.0081 | 0.1105 | -0.0553 | -0.0218 | 0.0146 | 0.0135 | 0.1070 | -0.0507 | Yes |

# Supplementary Table 4 The Selection Process of Covariates: Step 2 - Covariates Were Introduced into The Basic Model and Removed from The Complete Model to Observe the Change of the Regression Coefficient of X (X=extraversion, agreeableness, conscientiousness, nervousness, openness).

|  | Basic model | | | | | Complete model | | | | | | Selected | |
| --- | --- | --- | --- | --- | --- | --- | --- | --- | --- | --- | --- | --- | --- |
| Covariates | Extraversion | Agreeableness | Conscientiousness | Nervousness | Openness | Extraversion | Agreeableness | Conscientiousness | Nervousness | Openness |  | |  |
| Original coefficient | 0.0148 | -0.0253 | 0.0062 | 0.0148 | -0.0689 | 0.0614 | -0.0124 | -0.0253 | 0.0613 | -0.0028 |  | |  |
| Religion | 0.0148 | -0.0249 | 0.0063 | 0.0158 | -0.0689 | 0.0615 | -0.0125 | -0.0253 | 0.0611 | -0.0026 |  | |  |
| Political landscape | 0.0223* | -0.0331* | -0.0248* | 0.0024* | -0.0433* | 0.0609 | -0.0124 | -0.0245 | 0.0621 | -0.0036* | Yes | |  |
| Current work status | 0.0264* | -0.0427* | -0.0581* | -0.0034* | -0.0202* | 0.0583 | -0.0102* | -0.0146* | 0.0625 | -0.0100* | Yes | |  |
| Education level | 0.0164* | -0.0238 | 0.0011* | 0.0117* | -0.0489* | 0.0617 | -0.0148* | -0.0250 | 0.0605 | -0.0103* | Yes | |  |
| Chronic disease | 0.0200* | -0.0278 | -0.0009* | 0.0209* | -0.0625 | 0.0613 | -0.0108* | -0.0228 | 0.0618 | -0.0039* | Yes | |  |
| Household type | 0.0206* | -0.0181* | 0.0124* | 0.0204* | -0.0667 | 0.0603 | -0.0146* | -0.0263 | 0.0610 | -0.0015* | Yes | |  |
| Marital status | 0.0233* | -0.0427* | -0.0493* | -0.0003* | -0.0314* | 0.0607 | -0.0107* | -0.0199* | 0.0618 | -0.0052* | Yes | |  |
| Family income | 0.0183* | -0.0275 | 0.0045* | 0.0144 | -0.0635 | 0.0602 | -0.0115 | -0.0247 | 0.0614 | -0.0040* | Yes | |  |
| Negative event | 0.0214* | -0.0229 | 0.0208* | 0.0370* | -0.0784* | 0.0588 | -0.0054* | -0.0263 | 0.0515* | 0.0031* | Yes | |  |
| Depression | 0.0356* | 0.0076* | 0.0465* | 0.0637* | -0.0666 | 0.0440* | -0.0373* | -0.0503* | 0.0247* | -0.0084* | Yes | |  |
| Registered permanent residence | 0.0174 * | -0.0237 | 0.0065 | 0.0146 | -0.0609* | 0.0612 | -0.0130 | -0.0256 | 0.0616 | -0.0044* | Yes | |  |
